# Transmembrane transporter expression regulated by the glucosylceramide pathway in *Cryptococcus neoformans*

**DOI:** 10.1186/s13104-015-1613-y

**Published:** 2015-11-16

**Authors:** Arpita Singh, Antonella Rella, John Schwacke, Caterina Vacchi-Suzzi, Chiara Luberto, Maurizio Del Poeta

**Affiliations:** Department of Biochemistry and Molecular Biology, Medical University of South Carolina, Charleston, SC 29425 USA; Department of Medicine, Division of Infectious Diseases and International Health, University of Virginia, 345 Crispell Dr, Carter Harrison Building, Charlottesville, VA 22908 USA; Department of Molecular Genetics and Microbiology, Stony Brook University, 150 Life Science Building, Stony Brook, NY 11794 USA; Integrated Systems and Solutions Division, Scientific Research Corporation, Remount Road, North Charleston, SC 29406 USA; Department of Physiology and Biophysics, Stony Brook University, Stony Brook, NY 11794 USA; Department of Microbiology and Immunology, Medical University of South Carolina, Charleston, SC 29425 USA; Department of Craniofacial Biology, Medical University of South Carolina, Charleston, SC 29425 USA; Division of Infectious Diseases, Medical University of South Carolina, Charleston, SC 29425 USA; Department of Preventive Medicine, University of Stony Brook, Stony Brook, NY 11794 USA

**Keywords:** Microarray, Gene expression analyses, Gene-set enrichment, Transmembrane transporter, Glucosylceramide, Methylation

## Abstract

**Background:**

The sphingolipid glucosylceramide (GlcCer) and factors involved in the fungal GlcCer pathways were shown earlier to be an integral part of fungal virulence, especially in fungal replication at 37 °C, in neutral/alkaline pH and 5 % CO_2_ environments (e.g. alveolar spaces). Two mutants, ∆*gcs1* lacking glucosylceramide synthase 1 gene (*GCS1*) which catalyzes the formation of sphingolipid GlcCer from the C9-methyl ceramide and ∆*smt1* lacking sphingolipid C9 methyltransferase gene (*SMT*1), which adds a methyl group to position nine of the sphingosine backbone of ceramide, of this pathway were attenuated in virulence and have a growth defect at the above-mentioned conditions. These mutants with either no or structurally modified GlcCer located on the cell-membrane have reduced membrane rigidity, which may have altered not only the physical location of membrane proteins but also their expression, as the pathogen’s mode of adaptation to changing need. Importantly, pathogens are known to adapt themselves to the changing host environments by altering their patterns of gene expression.

**Results:**

By transcriptional analysis of gene expression, we identified six genes whose expression was changed from their wild-type counterpart grown in the same conditions, i.e. they became either down regulated or up regulated in these two mutants. The microarray data was validated by real-time PCR, which confirmed their fold change in gene expression. All the six genes we identified, viz siderochrome-iron transporter (CNAG_02083), monosaccharide transporter (CNAG_05340), glucose transporter (CNAG_03772), membrane protein (CNAG_03912), membrane transport protein (CNAG_00539), and sugar transporter (CNAG_06963), are membrane-localized and have significantly altered gene expression levels. Therefore, we hypothesize that these genes function either independently or in tandem with a structurally modified cell wall/plasma membrane resulting from the modifications of the GlcCer pathway and thus possibly disrupt transmembrane signaling complex, which in turn contributes to cryptococcal osmotic, pH, ion homeostasis and its pathobiology.

**Conclusion:**

Six genes identified from gene expression microarrays by gene set enrichment analysis and validated by RT-PCR, are membrane located and associated with the growth defect at neutral-alkaline pH due to the absence and or presence of a structurally modified GlcCer. They may be involved in the transmembrane signaling network in *Cryptococcus neoformans*, and therefore the pathobiology of the fungus in these conditions.

## Background

Cryptococcal meningitis is a leading cause of death in HIV patients [1]. It is caused by *Cryptococcus neoformans* (*Cn*), an environmental fungus commonly found in pigeon droppings and eucalyptus trees. Though it is commonly known for its ability to cause disease in immunocompromised individuals, it has recently been recognized as a causative agent of infection in immunocompetent individuals too [2, 3]. Amongst the fungal virulence factors identified, the most important are their ability to survive in the host environments (acid-neutral-alkaline pH, 37 °C, 5 % CO_2_), capsule formation, urease and melanin production (Reviewed in [4]).

Earlier reports had shown that the synthesis of fungal sphingolipids regulates the ability of the fungus to replicate in the host environments [5–7]. In particular, studies in two mutants (∆*gcs1* and ∆*smt1*) in the glucosylceramide (GlcCer) pathway, lacking glucosylceramide synthase 1 and C9 sphingolipid methyltransferase 1 gene respectively, had shown that these genes are essential for the fungus to replicate in neutral-alkaline pH, 37 °C, 5 % CO_2_ (the physiological conditions found in the alveolar microenvironment). Upon intranasal injection, these mutants do not replicate in the lung and, thus, they are not pathogenic in a mouse model of cryptococcal meningitis [6, 8, 9]. Both Δ*gcs1* and Δ*smt1* mutants do not migrate from the lung to the brain because they are trapped in a lung granuloma.

In addition to be required for pathogenicity of *Cn*, the GlcCer pathway is also essential for the pathogenesis of other human pathogens, such as *Candida albicans* [10–12] and *Aspergillus fumigatus* [13], and to plant pathogens, such as *Fusarium graminearum* [14]. Furthermore, the synthesis of GlcCer seems to be important during *Pneumocystis* pneumonia (PCP) as GlcCer synthase transcripts have been found to be abundant at the time of isolation of the fungus from fulminate lung infection [15], and for infection caused by dimorphic fungi, as GlcCer is detected only in the lung infective form (yeast) and not in the environmental form (mold) [16–18]. Taken together, these studies suggest that the GlcCer pathway is most likely a pan-fungal virulence pathway required in promoting fungal replication at 37 °C, in neutral/alkaline pH and 5 % CO_2_ environments (e.g. alveolar spaces), reviewed in [19].

GlcCer is a sphingolipid localized in cell membranes (mainly cell wall and plasma membranes) of *Cn* [20]. Structural studies had proposed the hypothesis that an alteration in the membrane lipid structure may result in an altered raft formation, thus affecting fungal membrane fluidity and rigidity [21, 22]. Thus given its specific location and function in promoting fungal cell replication in neutral-alkaline pH, 37 °C, and 5 % CO_2_, GlcCer may be involved, either directly or indirectly in anchoring specific membrane proteins essential for transferring key nutrients across the membranes necessary for cell cycle progression.

Upon inhalation of fungal cells into the lung, *Cn* will have to adapt and respond to a new temperature, a new pH and to a new concentration of CO_2_. Several studies have highlighted fungal responses to the 37 °C temperature [23], pH [24–26] and to CO_2_ [27]. These studies suggested that replication of *Cn* in these microenvironments requires maintenance of pH gradients across multiple membrane systems, regulation of inorganic carbon uptake and, most importantly, adjustment to changes in the abundances of different ions. In fact, transmembrane signaling complex have the potential to contribute to osmotic, pH and ion homeostasis [25, 26, 28]. Additionally the physical structure of the plasma membrane can also change upon cell exposure to a different environment [29, 30], resulting in activation, down-regulation or dislocation of transmembrane transporters. This hypothesis is supported by studies suggesting that a proper ratio of membrane sphingolipids and sterols is necessary to sustain the hydrophobicity of a transmembrane domain of certain channels regulating the transmembrane potential [31–33]. Thus, a change in the membrane framework occurring due to the changes in the composition and/or structure of membrane sphingolipids as reported earlier [9], could result in an alteration of membrane-spanning channels in these mutants.

In this study, we performed a transcriptional analysis of *C. neoformans* wild type, ∆*gcs1* and ∆*smt1* mutants grown at 37 °C, 5 % CO_2_ at either pH 4.0 or 7.2 ± 0.2. We then analyzed their gene expression profiles, focusing only on genes whose expression was significantly changed at pH 7.2 ± 0.2 versus pH 4.0 in both ∆*gcs1* and ∆*smt1* mutants but not in *C. neoformans* wild type and found six genes, all encoding for transmembrane transporters. Quantitative real-time PCR (RT-PCR) was used to confirm the changes in expression of these six genes found by the microarray studies.

## Methods

### Strains and media

*Cryptococcus neoformans* var. grubii serotype A strain H99 wild-type (WT), *C. neoformans* ∆*gcs1* mutant strain and *C. neoformans* ∆*smt1* mutant strain, both derived from strain H99, were used in this study. Cryptococcus strains were routinely grown at 30 °C and 0.04 % atmospheric CO_2_ in yeast peptone dextrose broth (YPD-1 % yeast extract, 2 % peptone, 2 % dextrose, BD). Dulbecco’s modified Eagle medium high glucose (DMEM high glucose), buffered with 50 mM HEPES, 10 % FBS (Fetal Bovine Serum) and 1 M sorbitol at pH 7.2 ± 0.2 or pH 4, were used as conditioned media for growing *C. neoformans* strains at 37 °C in presence of 5 % CO_2_.

### RNA isolation

Overnight YPD grown cultures of WT, ∆*gcs1* and ∆*smt1* mutant strain, were pelleted, washed twice with sterile Phosphate Buffered Saline (PBS) and inoculated in DMEM high glucose, buffered with 50 mM HEPES, containing 10 % Fetal Bovine Serum, 1 M sorbitol at pH 7.2 or pH 4 and shaken-incubated for 20 h at 37 °C, 5 % CO_2_. The cells were harvested by centrifugation at 5000*g* for 10 min and washed twice with PBS. The cell-pellets were flash-frozen in dry-ice/ethanol bath and stored at −80 °C until ready to use. Total RNA was extracted from *Cn* strains, as described previously [34]. Briefly, the cells were lyophilized overnight and 100 µl of lyophilized cells were transferred in 2 ml screw cap tubes and 1.25 ml of TRI reagent (Molecular Research Center, Inc.) was added. After homogenization, using Bead Beater 16, the tubes were incubated at room temperature for 10 min and centrifuged at 8000*g* to pellet cell debris and unbroken cells. The supernatants were transferred in fresh tubes along with 60 µl of BAN phase separation reagent (Molecular Research Center, Inc., Cincinnati, OH, USA). After mixing for 20–30 s and incubating at room temperature for 5 min, samples were centrifuged at 8000*g*. The aqueous phases were placed in fresh tubes along with 70 % ethanol, and loaded onto RNeasy isolation columns provided by RNeasy Mini Kit (Qiagen, CA, USA). Total RNA was further purified and eluted according to manufacturer’s instructions. Total RNA integrity was verified by Agilent 2100 Bioanalyzer (Agilent, Santa Clara, CA, USA). Concentration and sample purity were determined by Nanodrop ND-1000 (Nanodrop, DE, USA). Samples with 260/280 < 1.8, 260/230 **<** 1.8 were column purified and re-quantitated.

### Microarray experiment

Global gene expression changes due to different pH conditions during growth were assessed for WT, ∆*gcs1* and ∆*smt1* mutant strains on dual-channel Cy3-Cy5 *Cryptococcus neoformans* H99 Agilent microarray (Agilent, Santa Clara, CA, USA). The array design is available on GEO (platform accession number GPL13419). Microarray analysis was carried out at MOgene, LC (Saint Louis, MO, USA). Total RNA (1 µg) was direct labeled using Kreatech ULS, a RNA Labeling Kit for dual-color hybridization. WT control strain grown at pH 4.0 were labeled with Cy3 (green channel) and the mutants or WT grown at 7.2 ± 0.2 pH labeled with Cy5 (red channel). Concentrations and dye incorporation data (Cy3: 100–130 pmol/µg, Cy5: 70–100 pmol/µg) were determined by Nanodrop. After labeling, 300 ng of each sample were fragmented and hybridized to an Agilent custom anti-sense probe 8 × 15 K microarray (AMADID 019465) by manufacturer’s specifications with the following exception: 5 µl of Kreablock (included in the labeling kit) was added after fragmentation. Hybridization was carried out in a SureHyb chamber in an Agilent hybridization oven at 65 °C and 10 rpm for 17 h. Slides were washed using Agilent GE wash buffers and scanned on an Agilent C scanner at 5 µM. Data was extracted using Agilent Feature Extraction v. 10.7.1 software. The normalization method used for data extraction was Linear-Lowess. Raw data were expressed as Lowess-normalized log_2_ ratio (test/reference).

Experiments involving 15 microarrays for 5 conditions, conducted in triplicate, were used to compare WT to mutant expression at pH 4 and pH 7.2 ± 0.2. Entries in the associated data files included accession number, sequence description, and two intensity measurements, along with fold change estimate, and associated *p* value. Image processing, normalization, intensity estimation, and probe-level significance calculations were performed prior to this effort and data was taken from the files without additional processing.

### Quantitative real time PCR

cDNA was synthesized from 1 μg of the total RNA with SuperScript III RNase H-Reverse Transcriptase (Invitrogen) according to the manufacturer’s protocol. Real-time PCR was performed as described by Villani et al. [35] with some modifications. Briefly, 1 μl of cDNA, 1 μl each of forward and reverse primers from 10 μM stocks, 12.5 μl of Bio-Rad iQ SYBR Green 2 X Supermix and 9.5 μl of sterile water, were used for the qPCR reactions. All the reactions were performed in triplicate. Primer sequences were as follows: CNAG_02083 (Siderochrome-iron-transporter), forward: 5′-GCTTATCTTGCACTTACTGTCCTC-3′, reverse: 5′-TTTGGGAACAACGAGGTAGG-3′; CNAG_03912 (Membrane-protein), forward: 5′-GGTTTGCAAGGATTGATGCTTATC-3′, reverse: 5′-GTACGGTGGGTCAGAAAGGA-3′; CNAG_00539 (Membrane-transport-protein), forward: 5′-ACGCCGATGGATTATTACTAGC-3′, reverse: 5′-CGACCTAGCAGTTCCGAAAG-3′; CNAG_06963 (Sugar-transporter), forward: 5′-TGGTCCTATCGGTGACTACTCT-3′, reverse: 5′-GCCAACTTGAGACACAAGCA-3′; CNAG_05340 (Monosaccharide-transporter), forward: 5′-AGGGATCGTTAGCTTTTGGTATG-3′, reverse: 5′-GGAAAATTGTTCGAGGACGA-3′; CNAG_03772 (Glucose-transporter), forward: 5′-GGACTGGTGTCAACTTCATTTTC-3′, reverse: 5′-CGACACCAATGATACCGACA-3′; CNAG_00334 (Heat-shock-protein), forward: 5′-GAGTCGAGATCATCGCCAAC-3′, reverse: 5′-TTAACGTCAGCGTCGTCGTA-3′; CNAG_03780 (Prcdna95), forward: 5′-AAGGGTGTCGTTGCCTTCTAC-3′, reverse: 5′-ATAGCCAAAACCCTCCATCC-3′; CNAG_00092 (Mitochondrial-protein), forward: 5′-GACCCAAGACCATCTTCTTCT-3′, reverse: 5′-GCAGCGAGGGAGTAGTTGAC-3′; CNAG_01307 (Endoplasmic-reticulum-protein), forward: 5′-AGCGAAACTTGTACTTGACAGG-3′, reverse: 5′-GCCATACTCGGCATTCTGTT-3′; CNAG_01150 (δ-12-fatty-acid-desaturase), forward: 5′-CCTTGGCAGGTTCTCTCTTTCT-3′, reverse: 5′-GCGTTGTTGATGGCCTTACT-3′. The amplification reactions consisted of 1 cycle of 3 min at 95 °C, 40 cycles of 10 s at 95 °C and 45 s at 55 °C, one cycle of 1 min at 95 °C, one cycle of 1 min at 55 °C and 78 cycles of 10 s at 55 °C. The results were normalized to an internal control gene, actin. Primer sequence was as follows: CNAG_00483 (Actin), forward: 5′-ACATGTCTATGGAAGAAGAAGTCG-3′ reverse: 5′-ATACCGTGCTCAATGGGGTA-3′. The real-time PCR results were analyzed using Q-Gene^®^ software, which expresses data as the means of normalized expression.

## Results

### Microarray data analysis

The conditions and file names for this data are given below in Table 1. All data is MIAME compliant and the raw data has been deposited in a MIAME compliant database with a GEO accession number GSE69361, a full description and complete data sets are available at (http://www.ncbi.nlm.nih.gov/geo/query/acc.cgi?acc=GSE69361).

**Table 1:** Growth conditions of *C. neoformans* wild-type (WT) *∆gcs1* and *∆smt1* and corresponding microarray data file

Condition	Data file
WT vs *∆smt1* pH 7.2	MUSC DelPoeta 102309 WT7.2 Cy3 MUT17.2 Cy5_1
	MUSC DelPoeta 102309 WT7.2 Cy3 MUT17.2 Cy5_2
	MUSC DelPoeta 011410 WT7.2 Cy3 MUT17.2 Cy5
WT vs *∆gcs1* pH 7.2	MUSC DelPoeta 102309 WT7.2 Cy3 MUT27.2 Cy5_1
	MUSC DelPoeta 102309 WT7.2 Cy3 MUT27.2 Cy5_2
	MUSC DelPoeta 011410 WT7.2 Cy3 MUT27.2 Cy5
WT vs *∆smt1* pH 4	MUSC DelPoeta 102309 WT4.0 Cy3 MUT14.0 Cy5_1
	MUSC DelPoeta 102309 WT4.0 Cy3 MUT14.0 Cy5_2
	MUSC DelPoeta 011410 WT4.0 Cy3 MUT14.0 Cy5
WT vs *∆gcs1* pH 4	MUSC DelPoeta 102309 WT4.0 Cy3 MUT24.0 Cy5_1
	MUSC DelPoeta 102309 WT4.0 Cy3 MUT24.0 Cy5_2
	MUSC DelPoeta 011410 WT4.0 Cy3 MUT240 Cy5
WT pH 4 vs WT pH 7.2	MUSC DelPoeta 011410 WT4.0S1 Cy3 WTS1 Cy5
	MUSC DelPoeta 011410 WT4.0S2 Cy3 WTS2 Cy5
	MUSC DelPoeta 011410 WT40S3 Cy3 WTS3 Cy5

In this effort we seek to identify groups of genes that are differentially regulated with patterns that correlate with the differential replication rates of the *Cn* WT and mutant strains. Earlier efforts indicated similar growth rates for the WT and ∆*gcs1* or ∆*smt1* mutants at pH 4, but with significantly reduced replication rate for the mutants, but not the WT, at neutral-alkaline pH. Thus, we focused on the analysis of gene expression profiles that would be associated with the pH phenotype. The *Cn* genome was acquired from the *Cryptococcus grubii* H99 Database (http://www.broadinstitute.org/annotation/genome/cryptococcus_neoformans/MultiHome.html). Descriptions and PFAM identifiers for each probe on the array were extracted from the genome information file after mapping probe accession numbers to locus identifiers. Each probe was further annotated using Gene Ontology (GO) terms taken from the PFAM2GO mapping. Of the 6931 probes, 3338 were annotated with one or more GO terms. This subset and the associated GO terms became the basis for our gene set enrichment analysis. For gene selection and or identification, signature pattern together with regression based methods were used.

#### Method 1—signature patterns

A gene is declared a signature gene within a microarray experiment if its fold changes *p*-value fall below 0.01. Furthermore, a gene is declared a signature gene for a specific condition if it was found to be a signature gene in all replicates of experiments for that condition. Finally, a gene is considered a signature gene for a pattern of conditions if that gene is a signature gene for all conditions within the pattern and not a signature gene for any other conditions. Genes, therefore, are mapped into 1 of 16 possible patterns arising from the 4 comparisons (WT vs *∆smt1* at pH 4, WT vs *∆smt1* at pH 7.2 ± 0.2, WT vs *∆gcs1* at pH 4, WT vs ∆*gcs1* at pH 7.2 ± 0.2). For each pattern, gene set enrichment analysis was conducted. Of particular interest in this study are genes that are differentially expressed at pH neutral-alkaline pH but not at acidic pH, in both the mutants.

#### Method 2—regression

Concerned that signature gene selection criteria of Method 1 might be too restrictive, a regression-based analysis was also conducted. Observed log intensities are assumed to follow a linear model including array, pH, mutant, and gene effects and gene-pH, gene-mutant and gene-pH-mutant interactions. The regression model is given as

1 $$\log (I_{i,j,k} ) = a_{i} + p_{j} + m_{k} + g_{l} + \alpha _{l,j} + \beta_{l,k} + \gamma_{l,j,k}$$

with Intensity *I*_*i,j,k*_, effects for Array α_*i*_, pH p_*j*_, Mutant m_*k*_, and Gene g_*l*_ and interactions α_*l*__*,j,*_ β_*l,k,*_ and γ_*l,j,k*_. The model was fit using Least Absolute Shrinkage and Selection Operator (LASSO) as implemented in the R glmnet package [36] and the regression penalty parameter was chosen via cross-validation. Genes having non-zero gene-pH-mutant interactions for both mutants with effects in the same direction (coefficients having the same sign) were then selected for gene set enrichment analysis.

#### Gene set enrichment analysis

For genes mapped to each of the patterns under Method 1 (signature patterns) and for the selected genes from Method 2 (regression), gene set enrichment analysis was performed. Only genes within the subset of 3338 and, thus, annotated with GO terms were used in this analysis. Enrichment of GO-terms appearing within these groups of selected genes was assessed using the R topGO package version 1.16.0 [36] using the “classic” algorithm and “fisher” statistic. GO terms with p < 0.01 were considered significant.

A subset of six genes of interest, selected based on evidence of differential expression in the microarray studies and labeled with GO terms found to be significant in our gene set enrichment analysis, were analyzed further using qRT-PCR. Based on minimal differential expression across conditions and high signal levels, five additional genes were selected using the microarray data and were used as a reference. The collection of these 11 genes was subjected to RT-PCR for WT and mutants at pH 4 and pH 7.2 ± 0.2 (6 cases).

The relationship between observed cycle time at threshold, CT, was assumed to be related to initial amount, A, the negative inverse of the log amplification efficiency, S, and the intercept of the linear approximation, I through the following expression.

2 $${\text{CT }} = S \, \times \, log \, \left( A \right) \, + I$$

The reference amount against which all experiments were normalized was taken from the average of the log amounts of the five reference genes. As such, the log normalized expression for gene ‘g’ in strain ‘s’ under experimental condition ‘e’ (pH) is given by

3 $$\log (N_{s,e,g} ) = \log \left( {\frac{{A_{s,e,g} }}{{A_{ref,e,g} }}} \right) = \frac{1}{S}\left( {CT_{s,e,g} - \frac{1}{5}\sum {CT_{s,e,r} } } \right)$$

where the summation is over the five reference genes and A_ref,e,g_ is the average log amount of the reference genes. Of interest in this analysis are the four ratios comparable to the microarray experiments.

4 $$\begin{aligned} {\text{R}}_{g, 1} = { \exp }\left( {{ \log }\left( {N_{\varDelta smt1,4,g} } \right) - { \log }\left( {N_{WT,4,g} } \right)} \right) \hfill \\ {\text{R}}_{g, 2} = { \exp }\left( {{ \log }\left( {N_{\varDelta gcs1,4,g} } \right) - { \log }\left( {N_{WT,4,g} } \right)} \right) \hfill \\ {\text{R}}_{ g,3} = { \exp }\left( {{ \log }\left( {N_{\varDelta smt1,7,g} } \right) - { \log }\left( {N_{WT,7,g} } \right)} \right) \, \hfill \\ {\text{R}}_{ g,4} = { \exp }\left( {{ \log }\left( {N_{\varDelta gcs1,7,g} } \right) - { \log }\left( {N_{WT,7,g} } \right)} \right) \hfill \\ \end{aligned}$$

The model parameters S and I, the relative expressions for each experiment Ns, e,g and the ratios of interest R_g_, were inferred using methods from the Bayesian statistical framework [37]. Inferences were accomplished using JAGS (3.1.0) [38] through the rjags package (3.10) [39] [40] within R. All inferences were executed in a single step and CT measurements were assumed to share a common precision prior. A model burn-in of 50,000 samples preceded collection of 50,000 samples from which posterior credible intervals were determined.

A total of 381 genes were identified as signature genes in one or more of the conditions and 14 of the 16 possible signature patterns were associated with one or more signature genes (Table 2). All the genes mapped to their signature patterns were listed in additional file 1 DOI “10.6070/H48050MW“ at https://mynotebook.labarchives.com/share/Microarray/MjAuOHw5NDUwMy8xNi9UcmVlTm9kZS81ODE1Nzk3NTd8NTIuOA==. Of those 14 cases, 3 cases were found to have GO terms that were significantly enriched at the *p* < *0.01* levels. These include (1) only WT vs *∆smt1* at pH 4, (2) only WT vs *∆smt1* at pH 7.2 ± 0.2 and WT vs ∆*gcs1* at pH 7.2 ± 0.2, and (3) only WT vs ∆*gcs1* at pH 7.2 ± 0.2. In case 1, significant GO terms were associated with cellular lipid metabolism and included GO:0006650 glycerophospholipid metabolic process (p = 0.0022), GO:0030384 phosphoinositide metabolic process (p = 0.0022), GO:0046486 glycerolipid metabolic process (p = 0.0022), GO:0006644 phospholipid metabolic process (p = 0.0063), and GO:0019637 organophosphate metabolic process (p = 0.0073) and in case 3 significant terms included GO:0055114 oxidation reduction (p = 0.0016) and GO:0055085 transmembrane transport (p = 0.0025). Case 2, the case of greatest interest in this analysis, only GO:0055085 transmembrane transport (p = 0.0092) appeared significant with 4 of the 8 differentially expressed and annotated genes being labeled with this GO term.

**Table 2:** Signature patterns and number of genes identified in the experimental conditions

Differential	Relative	To	WT	Differential	Annotated
*Δsmt1* pH 4.0	*Δsmt1* pH 7.2	*Δgcs1* pH 4.0	*Δgcs1* pH 7.2	Genes	Sets
✓	✓			4	3
✓				37	15
✓		✓	✓	9	3
✓		✓		14	3
✓			✓	6	3
✓	✓		✓	2	2
✓	✓	✓	✓	6	1
	✓		✓	17	8
	✓			26	11
	✓	✓	✓	2	1
	✓	✓		0	0
		✓		50	16
		✓	✓	18	5
			✓	190	83

**Fig. 1:**
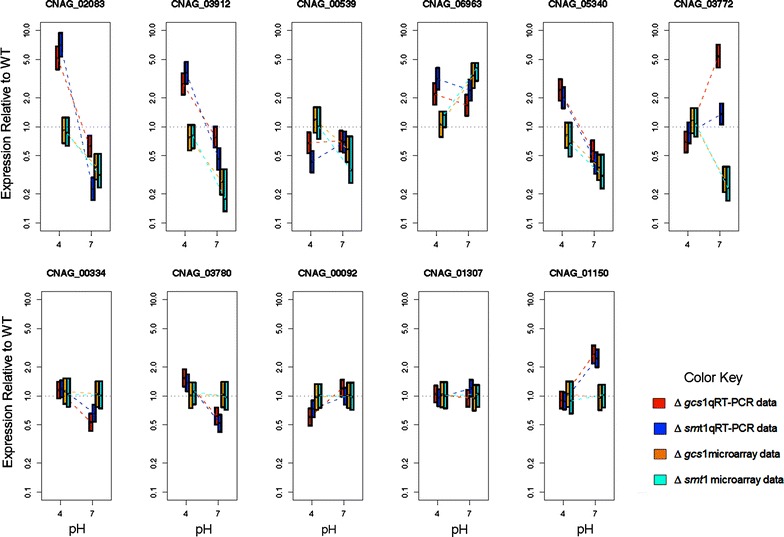
Results of microarray gene expression patterns validated by RT-PCR. The credible intervals for both the microarray (*orange and light blue*) and PCR (*red and dark blue*) results are for the six genes of interest and the five reference genes. The *orange*, *red* are results for ∆*gcs* *1*, *while light blue*, *dark blue* are for ∆*smt* *1* respectively. The six genes of interest are siderochrome-iron transporter (CNAG_02083), membrane protein (CNAG_03912), membrane transport protein (CNAG_00539), sugar transporter (CNAG_06963), monosaccharide transporter (CNAG_05340), glucose transporter (CNAG_03772). The five reference genes are heat shock protein (CNAG_00334), prcDNA95 (CNAG_03780), mitochondrial protein (CNAG_00092), endoplasmic reticulum protein (CNAG_01307), δ-12 fatty acid desaturase (CNAG_01150)

The regression-based analysis yielded similar findings. A total of 207 genes were found to have non-zero gene-pH-mutant interactions where the estimated coefficients were in the same direction for both mutants. Applying gene set enrichment analysis to the annotated subset of these genes identified 7 significantly enriched GO terms including GO:0055085 transmembrane transport (p = 3.3 × 10^−5^), GO:0055114 oxidation reduction (p = 0.00013) GO:0032787 monocarboxylic acid metabolic process (p = 0.00365), GO:0006810 transport (p = 0.00441), GO:0051234 establishment of localization (p = 0.00441), GO:0051179 localization (p = 0.00527), and GO:0008610 lipid biosynthetic process (p = 0.00574). Based on the results from both the signature and regression analyses, we selected 6 genes that were differentially expressed and labeled with GO:0055085 (transmembrane transport) including siderochrome-iron transporter (CNAG_02083), monosaccharide transporter (CNAG_05340), glucose transporter (CNAG_03772), membrane protein (CNAG_03912), membrane transport protein (CNAG_00539), and sugar transporter (CNAG_06963). The products of these genes are all part of the major facilitator superfamily (MFS) (PFAM PF07690.8) which are secondary carriers transporting small solutes in response to chemiosmotic ion gradients. As further confirmation of these findings, the set of six genes were analyzed by q-RTPCR. Quantitative real-time RT-PCR validated the results of microarray gene expression patterns. The credible intervals for both the microarray (orange and light blue) and PCR (red and dark blue) results are given in Fig. 1 for the six genes of interest and the five reference genes.

We see that siderochrome-iron transporter (CNAG_02083), membrane protein (CNAG_03912), and monosaccharide transporter (CNAG_05340), all exhibit expression that significantly exceeds the wild type in both mutants at pH 4 and expression significantly lower than that of the wild type at pH 7.2. The trend toward decreasing relative expression at pH 7.2 compared to pH 4 is consistent with the results of the microarray experiments. Interestingly, iron and/or glucose supplementation did not result in alteration of cell growth of the mutants Δ*gcs1*, Δ*smt1* at 37 °C, neutral/alkaline pH and 5 % CO_2_ environments in comparison to the *Cryptococcus* WT cultures (data not shown).

## Discussion

Fungi are notoriously known for their ability to survive in a diverse range of environmental conditions. Especially, mammalian pathogens survive inside the infected host by adapting themselves to the unique stress of various microenvironments by successfully linking the expression of virulence-associated phenotypes to host-derived pH and temperature cues [41]. *Aspergillus nidulans* and *Saccharomyces cerevisiae* have the PacC/Rim101 transcription factors mediating pH responses involving plasma membrane and endosomal signal complexes [24, 42]. In *Candida albicans*, Rim101 regulates the pH responsive pathway [43] involved in their transition from acidic to alkaline, which in turn stimulates a switch from yeast to filamentous form. Documented evidence of increase in GlcCer on the cell surface at alkaline pH in vitro [44] and also during infection, together with previous reports of GlcCer being associated with the ability to grow in alkaline pH, indicates therefore that this sphingolipid may play an important role in cryptococcal pH responsive pathway. In addition to PKA pathway being involved in tolerating increased pH in *Cryptococcus*, a rim101 mutant is hyper susceptible to not only elevated pH but also to iron deprivation [45].

In this study we have successfully used gene expression to identify genes differentially regulated in mutants deficient in a sphingolipid GlcCer. Our microarray studies identified six genes, all of which are involved in transporting ions or solutes across the plasma membrane. All of them have the conserved signature domain present in the Major Facilitator Superfamily members. CNAG_02083 or the siderochrome iron transporter has partial homology to MirA (siderphore transporter) of *Fusarium* and *Aspergillus* and to MFS of *Candida*. Similarly *Aspergillus* and *Candida* also have the partial homologue of the monosaccharide transporter (CNAG_05340), the glucose transporter (CNAG_03772) and the membrane transport protein (CNAG_00539), with no homologue as such in other fungi could be found for the membrane protein (CNAG_03912).

Membranes, being fluid, can sense changes in temperatures, pH, and atmospheric pressure. In a living cell, membranes thus, may react by changes in phase state and their microdomain organization. This in turn modulates intracellular signaling, which activates transcription, and thereby the activities of many membrane associated enzymes and transporters [46]. That these changes in protein activities are the direct read-outs of the expression of respective genes have been shown earlier in the case of heat shock genes [47] and also especially in many genes involved in lipid metabolism [48–50]. An important relevant example can be the yeast *PKC1* gene regulated pathway which is activated by “plasma –membrane stretch” [51] resulting from its increased fluidity under conditions of thermal stress. Given this scenario, it is reasonable to hypothesize that the changes in membrane dynamics of the two mutants *Δsmt1* and *Δgcs1* impacted by its physical structure have resulted in the changed level of transcription of certain transporters located on the membrane.

In *Cn*, major phenotypic changes and the expression of virulence factors are regulated by ion-uptake and homeostasis. More specifically, in addition to reduced fungal burden in mice brains infected with a mutant deficient in iron transporter, low iron induces capsule enlargement and represses laccase [52]. Cir1 considered as the master iron regulator controls the transcription of the genes involved in iron uptake and iron homeostasis and henceforth virulence in *Cn* [52]. The transcription of siderochrome iron transporter gene is modified in a *cir1* mutant, which eventually shows attenuation in virulence [53]. Altered iron and sugar homeostasis can result in a change in the melanin production, membrane trafficking and or copper or zinc loading. Our unpublished results demonstrating iron and/or sugar supplementation being unable to rescue the growth impairment in the mutants indicate that irreversible change/s in the physical structure of the membrane and hence some downstream signaling complex associated with the membrane may have happened in the two mutant strains Δ*gcs1* and Δ*smt1*. Thus, taken together, this may be a causal factor for impaired growth at this pH and hence attenuated virulence in these two mutants.

To facilitate comparison of the RT-PCR and microarray studies we sought a mathematical model that related the RT-PCR observations (measures of cycle time at threshold for each gene, condition, and strain) to the ratios observed in the microarray studies (R_g_, 1–4) for each of the genes of interest. Additionally, we sought to estimate those ratios in a manner that accounts for correlations in uncertainty when using common reference genes across our experimental conditions and strains using the wild type, under comparable conditions, as reference. To meet these objectives, we chose to express the relationships between the experimentally observed data and the ratios of interest in a single model and to use computational methods from the Bayesian statistical framework to make the required inferences. This process provided posterior density estimates for each of the ratios of interest and these results could then be directly compared to the microarray experiments (as shown in Fig. 1). The measures and the number of genes used in our reference set (5) are comparable to measures used to select genes developed by other commonly used software (e.g. geNorm which suggest 3–5). Similarly, the equations suggested by others (e.g. REST) are equivalent to those employed here. For confirmation of the reference gene as set selection, we plotted both microarray and RT-PCR expression changes and credible intervals for the reference set under variation in strain and pH in Fig. 1.

Some degree of variability in between our microarray data was found in the genes CNAG_00539 and CNAG_03772, though the RT-PCR data were consistent with the microarray data within credible intervals. Gene set enrichment analysis was advantageous over individual gene analysis as it detected subtle changes in expression, although verification of hits after gene-set enrichment analysis was only feasible because of a small subset of six genes identified. However, overall, in this study, in the use of microarray, we overcame the challenge of analyzing a huge amount of expression data, and simultaneously filter out the false-positives effectively by following a simple multi-pronged approach of selecting genes through gene set enrichment analysis on selected genes mapped through signature patterns along with a regression-based approach and their functional validation.

## Conclusion

Gene expression microarrays on WT and the mutants of the GlCer pathway identified a novel set of differentially regulated genes associated with the growth of *Cn* in neutral-alkaline pH, 37 °C, 5 % CO_2_. Using both signature gene selection method and a regression –based analytical model, gene set enrichment analysis finally zeroed-in on a subset of six genes: siderochrome-iron transporter, monosaccharide transporter, glucose transporter, membrane protein, membrane transport protein, and sugar transporter which were further validated by RT-PCR. These set of genes may therefore be involved in the down-stream transmembrane signaling network, connected to the GlcCer of the membrane, and therefore in-turn control the pathogen’s virulence—associated phenotype of growth defect in neutral-alkaline pH at 37 °C. Further studies will evaluate the role of these genes in the pathogenesis of *C. neoformans* and can answer specific mechanistic questions like how they are regulated when GlcCer is absent or its structure is altered.

## Availability of supporting data

The microarray data set supporting the results of this article are available in the Gene Expression Omnibus, at http://www.ncbi.nlm.nih.gov/geo/query/acc.cgi?acc=GSE69361.

The data set(s) supporting the results of this article listed in Table 2 is available as additional file 1 with DOI "10.6070/H48050MW" at https://mynotebook.labarchives.com/share/Microarray/MjAuOHw5NDUwMy8xNi9UcmVlTm9kZS81ODE1Nzk3NTd8NTIuOA==.

## Abbreviations

GlcCer: glucosylceramide
Cn: *Cryptococcus neoformans*
PCR: polymerase chain reaction
RT-PCR: quantitative real time-pcr
